# Sulfur Vacancies in ZnIn_2_S_4_ Boost Photocatalytic H_2_O_2_ Production: Unveiling the Role of Sulfur Vacancies in the Superoxide Radical Pathway for H_2_O_2_ Photosynthesis

**DOI:** 10.3390/molecules31091512

**Published:** 2026-05-02

**Authors:** Boyi Ma, Degang Li, Weimin Zhang, Siru Hao

**Affiliations:** School of Chemistry and Chemical Engineering, Shandong University of Technology, Zibo 255000, China; 19853352262@163.com (B.M.); wmzhang@sdut.edu.cn (W.Z.);

**Keywords:** zinc indium sulfide photocatalysts, sulfur vacancy, adsorption energy, H_2_O_2_ synthesis

## Abstract

Hydrogen peroxide (H_2_O_2_) is widely regarded as a clean and high-value chemical; however, its conventional industrial production remains both energy-intensive and environmentally unsustainable. In this study, sulfur-deficient ZnIn_2_S_4_ (denoted SDZIS) was developed as an efficient photocatalyst for H_2_O_2_ generation through oxygen reduction under visible-light irradiation. SDZIS photocatalysts with controllable sulfur-vacancy concentrations were synthesized via a one-step citric-acid-assisted hydrothermal process combined with NaOH etching. The results of transient photocurrent response and electrochemical impedance spectroscopy show that the separation efficiency of charge carriers has been improved. Compared with pristine ZnIn_2_S_4_, the optimized SDZIS catalyst achieved a nine-fold enhancement in the H_2_O_2_ production rate, reaching 2711.81 μmol g^−1^ h^−1^. Results of experimental and density functional theory calculations suggest that sulfur vacancies can modulate the catalyst work function and the adsorption energy of O_2_. Comparative experiments indicate that an appropriate concentration of sulfur vacancies can lead to a high H_2_O_2_ yield. Combined with scavenger tests, DMPO-EPR, and rotating ring disk electrode measurements, these results support a sulfur-vacancy-associated enhancement in charge separation and a tendency toward a superoxide-involved 2e^−^ ORR pathway for H_2_O_2_ production.

## 1. Introduction

Hydrogen peroxide (H_2_O_2_) is a green, high-energy oxidant widely used in the healthcare, chemical, and textile industries [1]. The market demand for H_2_O_2_ is substantial; however, the currently dominant anthraquinone-based synthetic method consumes excessive energy and generates substantial waste, whereas direct H_2_O_2_ synthesis requires expensive catalysts and poses explosion risks [2,3]. Therefore, there is an urgent need for safe, efficient, and economical methods of peroxide production.

The photocatalytic synthesis of H_2_O_2_ through the oxygen-reduction reaction (ORR) has attracted increasing research attention [4,5]. Various semiconductor materials, including g-C_3_N_4_, carbon dots (CDs) and In_2_S_3_, have been extensively investigated to enhance H_2_O_2_ yield [6,7,8,9]. However, the practical efficiency of traditional photocatalytic materials remains limited due to severe charge carrier recombination and insufficient active sites for oxygen adsorption [10]. Therefore, designing highly active photocatalysts to promote oxygen-reduction efficiency is of paramount importance for advancing sustainable H_2_O_2_ production.

In recent years, the ternary metal sulfide ZnIn_2_S_4_ has received increasing research attention [11,12]. The direct band gap of ZnIn_2_S_4_ is 2.4–2.7 eV, enabling the effective utilization of visible light. The unique layered structure expands the specific surface area and provides rapid pathways for charge-carrier transport. This synergistic combination markedly enhances the solar-to-chemical energy conversion efficiency of ZnIn_2_S_4_, highlighting its strong potential for practical photocatalytic applications [13].

During photocatalytic redox processes, the operation of ZnIn_2_S_4_ typically involves two main steps: (i) absorption of incident photons to generate electron-hole pairs and (ii) subsequent migration of conduction-band electrons to participate in reduction half-reactions, while valence-band holes move toward the surface to drive oxidation reactions [14,15]. In addition, ZnIn_2_S_4_ exhibits inherently low toxicity and can be synthesized from earth-abundant precursors, offering clear environmental and economic advantages compared with conventional photocatalysts [16].

Nevertheless, two intrinsic bottlenecks still hinder ZnIn_2_S_4_’s performance under practical conditions: rapid recombination of photogenerated carriers, which significantly reduces quantum efficiency, and sluggish surface oxidation kinetics, which limit the overall reaction rate [17]. To address these issues, considerable research efforts have been devoted to developing versatile modification strategies that accelerate charge-carrier separation and enhance surface reactivity in ZnIn_2_S_4_-based photocatalysts.

Multiscale modification strategies—including metal doping, heterointerface engineering, and defect/vacancy modulation—have been demonstrated to synergistically enhance the photocatalytic performance of ZnIn_2_S_4_ [18,19,20]. Liu et al. employed a phosphorus-doping strategy to tune the electronic structure of the Zn sites in ZnIn_2_S_4_, successfully switching the O_2_ adsorption configuration from side-on to end-on and markedly enhancing the activity and selectivity of photocatalytic H_2_O_2_ production [21]. Yao et al. constructed a Z-scheme ZnIn_2_S_4_/C-Bi_2_S_3_ heterojunction via a one-pot method, which significantly enhanced photocatalytic H_2_O_2_ production while simultaneously achieving efficient degradation of multiple pollutants, including tetracycline in wastewater [22]. Meanwhile, surface sulfur or zinc vacancies, and shallow defect states, act as ultrafast charge-trapping centers, anchoring photoexcited carriers and blocking nonradiative recombination pathways, thereby significantly enhancing the lifetime of charge carriers [23].

For instance, An et al. synergistically combined defect engineering with heterojunction construction to fabricate a sulfur-vacancy-rich TiO_2_/Vs-ZIS heterostructure, which markedly boosted photogenerated carrier separation and photocatalytic H_2_ evolution activity, delivering a hydrogen production rate of 17,113 μmol g^−1^ h^−1^—three times that of pristine Vs-ZIS [24]. Zhao et al. synthesized a ZnIn_2_S_4_ photocatalyst rich in sulfur vacancies via a hydrothermal-calcination route. The vacancies enhanced visible-light absorption and oxygen-evolution kinetics, promoting the efficient separation of photogenerated electron-hole pairs and achieving greater than 93% degradation of 50 µg L^−1^ PFOA in surface water and aquaculture wastewater [25]. Huang et al. introduced sulfur vacancies into ZnIn_2_S_4_ by calcination in air; the vacancies drastically modify the coordination environment and tune the catalyst’s adsorption strength toward intermediates, enabling a H_2_O_2_ evolution rate of 1706.4 μmol g^−1^ h^−1^ under visible-light irradiation without any sacrificial agent [26]. Wei et al. employed a low-temperature water-bath method to precisely engineer tunable concentrations of Zn-S dual vacancies within ZnIn_2_S_4_ nanosheets, doubling the sacrificial-agent-free photocatalytic H_2_O_2_ production rate and achieving twice the activity of the unmodified material [27]. Although previous studies have established ZnIn_2_S_4_ as a highly efficient photocatalyst, the controllable creation of vacancies via defect engineering remains largely unexplored. Moreover, the fine-tuning of catalytic performance by varying the sulfur-precursor dosage remains to be systematically investigated.

Herein, we employed a simple one-step hydrothermal route using citric acid (CA) and NaOH as additives, to vary the sulfur-precursor dosage systematically and directly grow a series of sulfur-deficient ZnIn_2_S_4_ atomic layers. The SDZIS exhibits high catalytic performance and robust stability for H_2_O_2_ production under visible light, with a H_2_O_2_ production rate of 2711.81 μmol g^−1^ h^−1^, and retaining more than 90% of the initial activity after four cycles. We reveal that the introduced sulfur vacancies create a dual optimization effect: increasing photogenerated carrier formation, as indicated by the enhanced photocurrent response and suppressing electron–hole recombination, as confirmed by Photoluminescence Spectroscopy (PL) results and improving both ORR selectivity and catalytic activity, leading to a higher H_2_O_2_ production rate. Density functional theory (DFT) calculations further show that these vacancies lower O_2_ adsorption energy, promote O_2_ desorption to •O_2_^−^, and thereby accelerate H_2_O_2_ formation via a homogeneous-like pathway. Theoretical and experimental results demonstrate that defect engineering, which introduces abundant sulfur vacancies during NaOH etching, secures a high density of active sites, along with enhanced charge density and mobility, thereby markedly boosting visible-light-driven H_2_O_2_ production. Although sulfur-vacancy engineering in ZnIn_2_S_4_ has been reported previously, a clearer correlation between vacancy-induced electronic modulation, O_2_ activation tendency, and H_2_O_2_ photosynthesis remains desirable. In this work, we employ a CA/NaOH-assisted hydrothermal route to prepare sulfur-deficient ZnIn_2_S_4_ and investigate how sulfur-vacancy-related surface/electronic changes correlate with photocatalytic H_2_O_2_ production.

## 2. Results

### 2.1. Morphology and Microstructure Analysis

**Figure 1:**
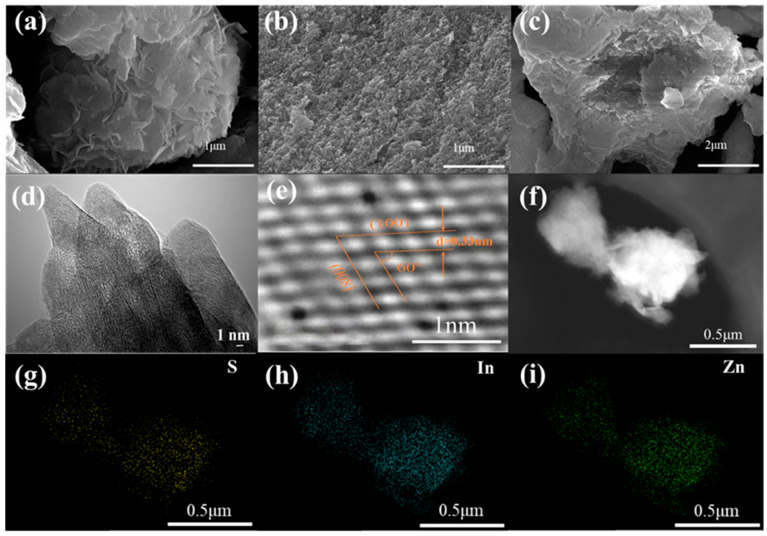
SEM images of (**a**) ZIS, (**b**) SDZIS, and (**c**) SDZIS with excess sulfur addition; (**d**,**e**) HRTEM and (**f**–**i**) the corresponding EDS element S, In and Zn mappings of the SDZIS.

**Figure 2:**
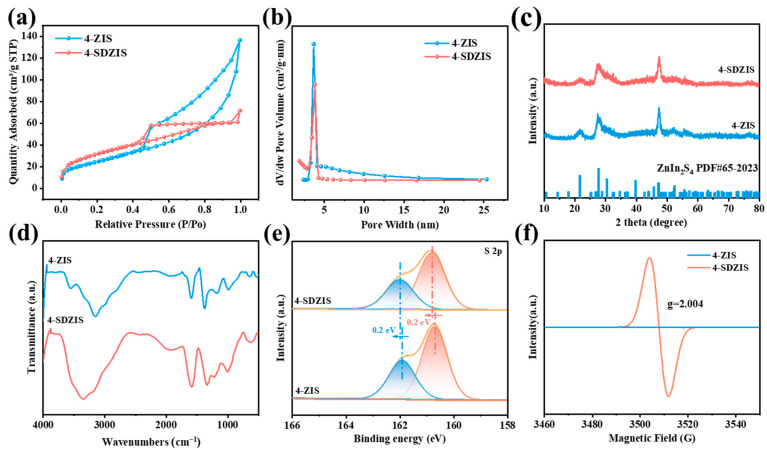
(**a**) Nitrogen adsorption–desorption isothermal curve; (**b**) Pore size distribution curves. (**c**) XRD patterns, (**d**) FT-IR spectra, (**e**) S 2p XPS spectra, and (**f**) EPR spectrum of 4-ZIS and 4-SDZIS.

We used a scanning electron microscope (SEM) and a high-resolution transmission electron microscope (HRTEM) to examine the surface morphology of the obtained samples systematically. Figure 1a shows that the one-step hydrothermal route produced ZIS as large, stacked nanosheets. The combined presence of CA and NaOH transformed the stacked ZIS nanosheets into much finer and thinner SDZIS nanosheets (Figure 1b). Specifically, CA acts as a chelating agent that retards crystal growth and NaOH serves as an alkaline surface etchant that induces surface reconstruction and promotes the formation of sulfur-deficient sites. Under the same CA/NaOH conditions, increasing the TAA dosage gradually weakened the etch: 8 mmol TAA left only partial etching and retained large nanosheets (Figure 1c). HRTEM images (Figure 1d,e) further reveal that SDZIS nanosheets become markedly thinner and smaller than pristine ZIS. This structural refinement enlarges the specific surface area and supplies more accessible reactive sites. The lattice fringe spacing of 0.33 nm corresponds well to the d(100) plane, and the measured 60° dihedral angle agrees with the calculated angle between the (100) and (008) planes of hexagonal ZnIn_2_S_4_ [28]. Energy-dispersive spectroscopy (EDS) elemental mapping (Figure 1f) demonstrated the uniform distribution of S, In and Zn throughout the catalyst. Suggesting sulfur depletion after CA/NaOH treatment. Considering that EDS is semi-quantitative, the formation of sulfur vacancies is more reliably supported by X-ray photoelectron spectroscopy (XPS) (Figure 2e) and electron paramagnetic resonance (EPR) (Figure 2f) analyses.

### 2.2. Surface Chemical State and Structure Analysis

Figure 2a,b show the nitrogen adsorption–desorption isotherms and corresponding pore-size distribution curves of the photocatalytic materials. Both samples exhibited typical type IV isotherms, confirming the existence of mesoporous structures. The BET-specific surface areas of 4-ZIS and 4-SDZIS were 90.36 m^2^/g and 114.30 m^2^/g, respectively, which indicate that treatment of ZIS with CA and NaOH markedly increases the specific surface area, thereby providing more accessible active sites [29]. However, considering that the specific surface area increases only from 90.36 to 114.30 m^2^ g^−1^, the increase in catalyst surface area was not significant enough; thus, the surface area gain from etching should not be the primary reason for the improvement in catalyst performance. Therefore, the dominant contribution should be attributed to other factors, such as sulfur-vacancy-induced electronic modulation, rather than surface-area enlargement alone.

Figure 2c presents the XRD patterns of 4-ZIS and 4-SDZIS, which can be indexed to the hexagonal phase of ZnIn_2_S_4_ (JCPDS card no. 65-2023). The diffraction peaks of SDZIS did not change compared with those of ZIS. Furthermore, varying the sulfur-precursor amount did not cause a noticeable shift or change in intensity in the XRD patterns (Figure S1), indicating that the modification process exerted a negligible influence on the overall crystal structure. In addition, the Fourier-transform infrared (FT-IR) spectra (Figure 2d) further confirmed the structural similarity between ZIS and SDZIS.

As shown in Figure 2e, the S 2p XPS spectrum of 4-SDZIS shows a positive shift (~0.2 eV) relative to that of 4-ZIS, indicating a reduced electron density around sulfur atoms, attributed to the formation of sulfur vacancies [30]. Introducing sulfur vacancies disrupts the local coordination environment and weakens electron screening, leading to higher binding energies. This electronic modulation is consistent with the enhanced charge separation and improved photocatalytic activity of 4-SDZIS. Based on the available XPS data, we have additionally performed a semi-quantitative estimation of the sulfur vacancy concentration in the optimized sample, and the results are presented in Table S1. In addition, the binding energies of Zn 2p and In 3d in Figure S2 show negligible differences between 4-ZIS and 4-SDZIS, suggesting that the electronic environments of Zn and In remained largely unchanged. These results suggest that sulfur atoms may act as active sites in the oxygen-reduction reaction (ORR) to produce hydrogen peroxide. The presence of abundant sulfur vacancies in SDZIS provides more unsaturated sites capable of producing lone-pair electrons, thereby accelerating the photocatalytic formation of H_2_O_2_. To further verify the presence and influence of vacancies, electron paramagnetic resonance (EPR) analysis was conducted, as shown in Figure 2f. Compared with 4-ZIS, 4-SDZIS exhibited a much stronger EPR signal. A stronger EPR intensity corresponds to a higher concentration of unpaired electrons, confirming the existence of sulfur vacancies [31]. This observation is consistent with the XPS results.

### 2.3. Optical and Photoelectrochemical Properties

To elucidate the charge-separation mechanism in SDZIS, we performed a series of complementary characterization analyses. Generally, the photocatalytic oxygen-reduction reaction (ORR) for hydrogen-peroxide generation proceeds through three main steps: (i) photoexcitation to produce electron-hole pairs; (ii) adsorption of O_2_ molecules on the catalyst surface to generate superoxide radicals (•O_2_^−^); and (iii) subsequent combination of these radicals with protons in the solution to yield H_2_O_2_.

**Figure 3:**
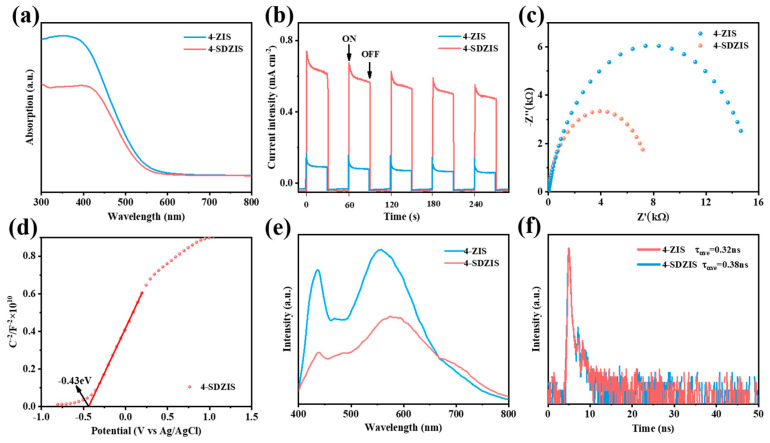
(**a**) UV-vis absorption spectra; (**b**) Transient photocurrent response; (**c**) EIS spectra of as-prepared samples under visible-light illumination; (**d**) Mott–Schottky (M-S) plot of 4-SDZIS; (**e**) Steady state photoluminescence spectroscopy (PL); (**f**) Time-resolved photoluminescence spectroscopy (TRPL).

The influence of sulfur vacancies on the band structure was investigated by ultraviolet-visible diffuse reflectance spectroscopy (UV-Vis DRS), and the results are shown in Figure 3a. Compared with 4-ZIS, the sulfur-vacancy-rich 4-SDZIS exhibited a distinct blue shift in its absorption edge, indicating enhanced electron-donating capability. The calculated bandgap energies (Eg) were 2.52 eV for 4-ZIS and 2.49 eV for 4-SDZIS. Mott-Schottky (M-S) measurements were further used to determine the flat-band potentials (Efb) by extrapolating the linear regions of the curves (Figure 3d). The positive slopes confirm that both materials are n-type semiconductors. The Efb values for 4-ZIS and 4-SDZIS were determined to be −0.24 eV and −0.43 eV, respectively (vs. Ag/AgCl). For n-type semiconductors, the conduction-band potential (ECB) is typically ~0.1 eV more negative than the Efb. Accordingly, the ECB values of 4-ZIS and 4-SDZIS were estimated as −0.14 eV and −0.33 eV, respectively. Based on the relationship Eg = E_VB_ − E_CB_, the corresponding valence-band potentials (E_VB_) were calculated to be 2.38 eV and 2.16 eV, respectively (Table S2).

Notably, the efficient initiation of the oxygen reduction reaction (ORR) in this system critically depends on the effective separation and rapid migration of photogenerated charge carriers [32]. Specifically, photoexcited electron-hole pairs undergo directional transport from the bulk semiconductor to surface active sites, a process essential for enhancing H_2_O_2_ production efficiency. Electrochemical characterization provided further insight into the charge-separation behavior of the ZnIn_2_S_4_-based materials.

As shown in Figure 3b, the transient photocurrent response measurements reveal that 4-SDZIS exhibited a photocurrent density approximately three to four times higher than that of 4-ZIS during the 30 s light on/off cycles, indicating substantially improved visible-light photoresponse. Furthermore, the charge-transfer properties of the photocatalysts were evaluated using electrochemical impedance spectroscopy (EIS), and the results are presented in Figure 3c. The Nyquist plots show that 4-SDZIS possesses a markedly smaller semicircular radius than 4-ZIS, indicating a reduced charge-transfer resistance after modification. This result is consistent with the transient photocurrent behavior, confirming the superior charge-separation efficiency and enhanced interfacial electron transport in 4-SDZIS.

From the perspective of photocatalytic H_2_O_2_ generation, the Mott-Schottky (M-S) plots (Figure 3d and Figure S3) reveal distinct differences in the band structure and charge-separation behavior between 4-ZIS and 4-SDZIS. The flat-band potential of 4-ZIS was approximately −0.24 eV (vs. NHE), suggesting a relatively negative conduction-band position that confers moderate reducing power and facilitates the participation of photogenerated electrons in the O_2_ reduction process for H_2_O_2_ formation. However, the shallower slope of the M-S curve indicates a lower carrier concentration, which likely limits electron-hole separation and, consequently, suppresses H_2_O_2_ productivity. In contrast, the flat-band potential of 4-SDZIS shifted further negatively to −0.43 eV (vs. NHE), implying enhanced reducing capability that favors multi-electron transfer pathways for H_2_O_2_ generation. Moreover, the steeper slope of its M-S curve indicates that sulfur vacancies significantly increase the carrier density, thereby enhancing charge separation and improving photocatalytic performance.

Steady-state photoluminescence spectroscopy further evaluated the recombination behavior of photogenerated carriers. As shown in Figure 3e, 4-SDZIS exhibited a substantially lower PL emission intensity than 4-ZIS, strongly suggesting that sulfur vacancies effectively suppress the radiative recombination of photoinduced electron-hole pairs. Complementary time-resolved photoluminescence (TRPL) spectra (Figure 3f) revealed similar emission profiles for both samples. The average lifetime (τ) of 4-SDZIS was 0.38 ns, slightly longer than that of 4-ZIS (τ = 0.32 ns), with only a minor difference (Δτ = 0.06 ns). This negligible variation indicates that the improvement in photocatalytic activity does not primarily arise from prolonged carrier lifetime. Rather, these TRPL findings, together with the PL results, confirm that the enhanced H_2_O_2_ generation efficiency of 4-SDZIS originates from more efficient charge separation and suppressed carrier recombination driven by sulfur-vacancy modulation.

## 3. Discussion

### 3.1. Photocatalytic Performance

**Figure 4:**
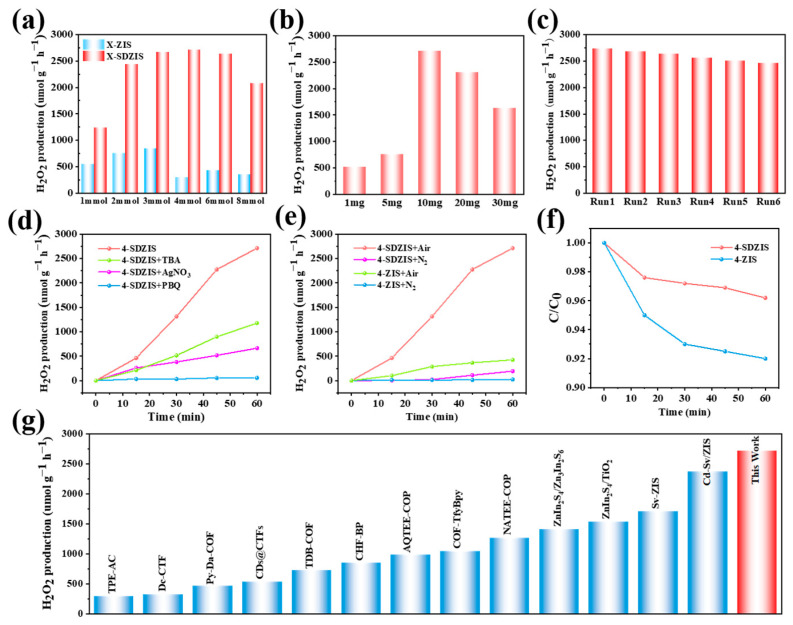
(**a**) Comparison of photocatalytic activity between X-ZIS and X-SDZIS; (**b**) H_2_O_2_ production rates with varying catalyst amounts; (**c**) H_2_O_2_ evolution cycling stability test; (**d**) H_2_O_2_ yield with various radical scavengers; (**e**) Activity comparison under different gas conditions; (**f**) Decomposition of H_2_O_2_ over time; (**g**) Comparison of H_2_O_2_ release rates of various recently reported catalysts.

Comprehensive characterization results confirmed that SDZIS exhibits significant potential as a photocatalyst for H_2_O_2_ generation from H_2_O and O_2_. We adjusted the TAA amount to synthesize a series of photocatalysts with varying sulfur concentrations, thereby elucidating the effect of sulfur content on photocatalytic performance. These samples were denoted as X-ZIS and X-SDZIS, where X corresponds to the TAA dosage in mmol. Systematic evaluation of their photocatalytic activities (Figure 4a) revealed that the H_2_O_2_ production efficiency initially increased with TAA dosage, reaching a maximum before declining at higher sulfur contents. Among all tested samples, 4-SDZIS exhibited the highest catalytic performance, achieving an outstanding H_2_O_2_ yield of 2711.81 μmol g^−1^ h^−1^, approximately nine times greater than that of 4-ZIS. These findings led us to select 4-ZIS and 4-SDZIS as the representative samples for subsequent in-depth investigations.

Figure 4b shows the influence of different 4-SDZIS catalyst loadings on H_2_O_2_ production. The H_2_O_2_ generation efficiency exhibits a distinct trend: initially increasing and then decreasing with increasing catalyst amount. The system achieves optimal catalytic performance at a catalyst loading of 10 mg, with a maximum H_2_O_2_ production rate of 2711.81 μmol g^−1^ h^−1^, significantly outperforming other tested conditions. The H_2_O_2_ production rate retained approximately 90% of its initial value after six consecutive cycles (Figure 4c), indicating excellent catalytic durability. To assess the structural stability of the catalyst, we performed XRD characterization on the recycled 4-SDZIS sample after centrifugation and drying. As shown in Figure S4, the XRD patterns of the recycled catalyst exhibit no noticeable changes compared with the fresh sample, confirming the outstanding structural stability of the sulfur vacancy-rich 4-SDZIS.

To test the long-term operational stability and integrity of the catalyst, we conducted a long-term test, as shown in Figure S5. The catalyst’s activity rose rapidly within the first few hours and reached a stable plateau. Subsequently, during the 30 h continuous operation, it only showed a slight decrease. The maximum H_2_O_2_ production rate was approximately 2725 μmol g^−1^ h^−1^, and it still maintained around 2645 μmol g^−1^ h^−1^ at 30 h, corresponding to an activity retention rate of approximately 97%, indicating that under the current reaction conditions, the catalyst can maintain a high and relatively stable catalytic activity during the continuous operation for several tens of hours without showing any significant deactivation.

We further compared the photocatalytic H_2_O_2_ production performance in the presence and absence of a sacrificial agent (Figure S6). The results showed that in the ethanol-containing system, the catalyst exhibited higher H_2_O_2_ production activity. In contrast, in the ethanol-free and pure water conditions, the system still detected significant H_2_O_2_ production, indicating that the catalyst itself has a certain ability to perform photocatalytic H_2_O_2_ production without sacrificial agents. However, the yield under the ethanol-free condition was significantly lower, only a fraction of that in the ethanol-containing system, indicating that ethanol indeed plays an important role as a hole-sacrificing agent in the current system, helping to inhibit the recombination of photogenerated carriers and promoting electron participation in the O_2_ reduction reaction.

**Scheme 1:**
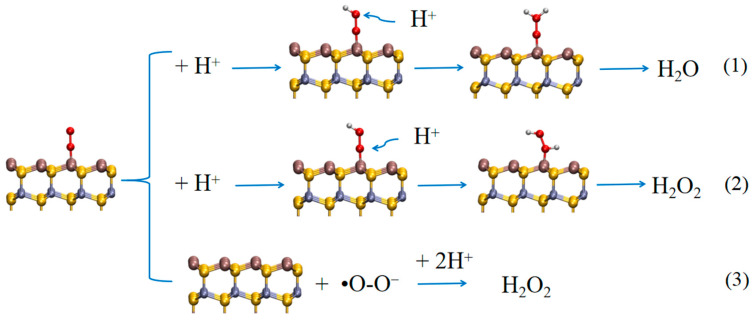
Schematic diagram of the oxygen reduction process.

To further explore the factors influencing H_2_O_2_ generation, we evaluated the effects of various radical scavengers on the catalytic activity (Figure 4d). The addition of p-benzoquinone (PBQ, a •O_2_^−^ scavenger) completely suppressed H_2_O_2_ formation, indicating that •O_2_^−^ is a key reaction intermediate. Moreover, the presence of AgNO_3_ (an electron sacrificial agent) significantly reduced the H_2_O_2_ production, suggesting that the photo-generated electrons transfer to adsorbed O_2_, leading to the formation of •O_2_^−^. Subsequently, •O_2_^−^ reacts sequentially with two H^+^ ions, ultimately generating H_2_O_2_. The conventional photocatalytic production of H_2_O_2_ can thus be described by Scheme 1:

In general, the oxygen reduction reaction (ORR) proceeds via a four-electron pathway to produce water. Initially, oxygen molecules are adsorbed onto the catalyst surface. The adsorbed oxygen then accepts one electron and reacts with a proton (H^+^) from the solution to form an adsorbed *OOH intermediate. If the second H^+^ tends to attack the topmost oxygen atom, as shown in reaction process (1) of Scheme 1, *O-OH_2_ (* represents adsorption on the catalyst surface) will be generated, and one molecule of water will be quickly removed. In addition, certain catalysts can increase the electronegativity of oxygen atoms directly bonded to the catalyst surface. In this scenario, the second H^+^ preferentially adsorbs onto these oxygen atoms (reaction step 2 in Scheme 1), leading to the formation of an adsorbed H_2_O_2_ intermediate. The intermediate desorbs, producing free H_2_O_2_.

**Scheme 2:**
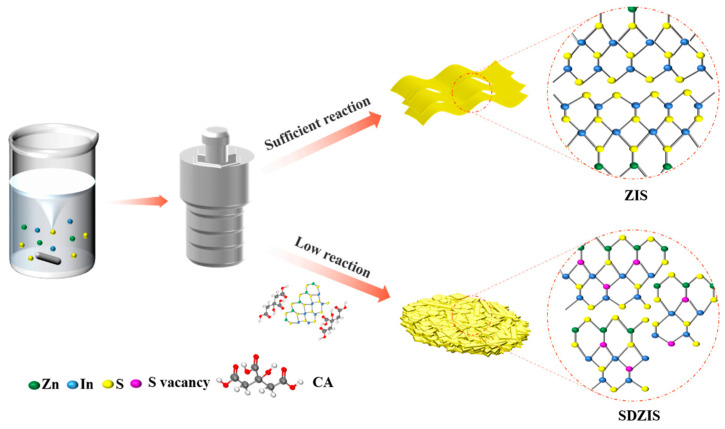
Schematic diagram of the preparation of ZIS and SDZIS.

Scheme 1 route (3) illustrates the plausible pathway for H_2_O_2_ formation. The relatively weak O_2_ adsorption energy on SDZIS (−0.33 eV) enables adsorbed O_2_ to desorb readily and form free •O_2_^−^ after electron transfer, accounting for this behavior. A detailed comparison of the three reaction pathways in Scheme 2 suggests that pathway (3) resembles a reaction mode in which O_2_ first accepts an electron at the catalyst interface and then forms desorbed •O_2_^−^ species that further react in solution, rather than remaining exclusively as surface-bound intermediates throughout the entire pathway. As is widely recognized, homogeneous reactions generally exhibit higher rates than heterogeneous processes, which may explain the efficient generation of H_2_O_2_ observed over SDZIS.

Furthermore, as shown in Figure 4d, the addition of tert-butanol (TBA, a •OH scavenger) reduces the H_2_O_2_ yield by approximately 50%. This effect is likely associated with the decomposition equilibrium of H_2_O_2_, which produces hydroxyl radicals. Consequently, TBA promotes the decomposition of H_2_O_2_, thereby lowering the observed yield.

Given the critical role of O_2_ adsorption in H_2_O_2_ photosynthesis, the photocatalysts’ catalytic activity was evaluated under different gas atmospheres. As shown in Figure 4e, continuous N_2_ bubbling led to a pronounced decrease in H_2_O_2_ production for both 4-ZIS and 4-SDZIS, demonstrating the indispensable role of O_2_ in the oxygen reduction process. The stability of H_2_O_2_ in the presence of the two catalysts was also investigated, with the results summarized in Figure 4f. The decomposition rate of H_2_O_2_ over 4-SDZIS is significantly lower than that over 4-ZIS, indicating that sulfur vacancies effectively suppress H_2_O_2_ decomposition.

Figure 4g compares the photocatalytic H_2_O_2_ production rate of 4-SDZIS with those of several recently reported advanced materials to highlight its superior catalytic activity. The results show that 4-SDZIS exhibits higher photocatalytic performance than previously reported systems under comparable conditions, including covalent organic frameworks (COFs) [33,34], covalent triazine frameworks (CTFs) [35], covalent organic polymers (COPs) [36], and other indium zinc sulfide-based photocatalysts. Moreover, indium-zinc-sulfide-based materials generally exhibit superior overall photocatalytic performance compared with other classes of materials, further underscoring their broad potential in photocatalysis [37,38,39].

### 3.2. Mechanism of the Enhanced Performance

From a mechanistic perspective, the O_2_ reduction reaction (ORR) proceeds via two single-electron transfer steps: O_2_ first accepts one electron to form •O_2_^−^, which subsequently acquires another electron and two protons (H^+^) to yield H_2_O_2_. To investigate this process, the electron transfer number (n) was monitored in an O_2_-saturated 0.1 M KOH electrolyte. Figure S7 displays the RRDE polarization curves of 4-ZIS and 4-SDZIS at a rotation rate of 1600 rpm, showing the ring current (top) and disk current (bottom) [40,41]. As the potential shifts negatively from 0 V (vs. Ag/AgCl), the disk current gradually increases, while both 4-ZIS and 4-SDZIS exhibit similar trends in ring current variation. Notably, 4-SDZIS displays a significantly higher ring current than 4-ZIS, indicating that the abundant sulfur vacancies in SDZIS substantially enhance H_2_O_2_ generation.

**Figure 5:**
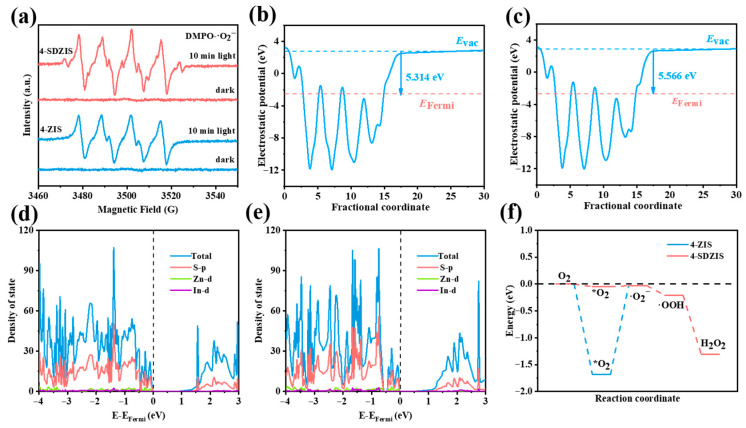
(**a**) EPR of DMPO-•O_2_^−^ for 4-ZIS and 4-SDZIS; (**b**,**c**) The work function diagrams of 4-SDZIS and 4-ZIS; (**d**,**e**) PDOS plots of 4-SDZIS and 4-ZIS; (**f**) The Gibbs free energy reaction step diagrams of 4-ZIS and 4-SDZIS. “*” refers to the adsorbent (catalyst).

Furthermore, Figure S8 presents the corresponding n values in the potential range of −1 to −0.6 V (vs. Ag/AgCl), which approach 2, confirming that the ORR follows a two-electron pathway. Electron paramagnetic resonance (EPR) trapping experiments using 5, 5-dimethyl-1-pyrroline N-oxide (DMPO) were conducted to verify the presence of •O_2_^−^ in the system [42,43]. The EPR results reveal that 4-SDZIS exhibits a stronger DMPO-•O_2_^−^ signal than 4-ZIS (Figure 5a), indicating a more pronounced capacity for a higher concentration of •O_2_^−^ generation. Consistent with the discussion in Scheme 2, the abundant •O_2_^−^ facilitates a faster increase in H_2_O_2_ yield via a homogeneous catalytic pathway.

In DFT calculations, the work function is a fundamental physical parameter that reflects the ease with which electrons can escape from a material’s surface, which defines the minimum energy required to transfer an electron from the interior of the material (near the Fermi level) to the vacuum level [44]. Consequently, a lower work function is favorable for catalytic reactions. Figure 5b,c show the calculated work functions of 4-SDZIS and 4-ZIS, respectively. Notably, 4-SDZIS exhibits a significantly lower work function (5.314 eV) compared with 4-ZIS (5.566 eV), facilitating electron escape from the catalyst surface and lowering the energy barrier for electron transfer to adsorbed O_2_, thereby promoting the formation of •O_2_^−^ intermediates. This reduced work function, therefore, directly contributes to the enhanced two-electron oxygen reduction pathway toward H_2_O_2_ formation.

As shown in Figure S9, the work functions of catalysts with different sulfur vacancies can also be observed. As the number of sulfur vacancies increases, the absolute value of the work function decreases, indicating that the increase in sulfur vacancies is beneficial for the escape of electrons from the catalyst, thereby facilitating the transfer of electrons to the adsorbed O_2_ (Figure S11). However, when the number of sulfur vacancies reaches 3, the work function begins to increase, indicating that too many sulfur vacancies are not conducive to electron loss by the catalyst, resulting in low catalytic activity. As shown in Figure S10, with increasing S vacancy concentration, the adsorption energy of O_2_ gradually decreases. When Vs = 3, the adsorption energy becomes positive, indicating that no more O_2_ will be adsorbed at this time; that is, the appropriate S vacancies can promote the conversion of O_2_ into superoxide radicals, but too many S vacancies are actually not conducive to the adsorption of O_2_. Therefore, appropriately distributed sulfur vacancies can enhance the efficiency of photocatalytic H_2_O_2_ production.

Moreover, a reduced work function is likely associated with higher photocurrent and more efficient charge separation, consistent with the experimental observation in Figure 3b, where 4-SDZIS displays a stronger photocurrent response. Additionally, the introduction of sulfur vacancies generates mid-gap states, effectively narrowing the bandgap. This modification enhances visible-light absorption, further boosting the efficiency of photocatalytic H_2_O_2_ production [45].

Figure 5d,e present the results of projected density of states (PDOS) calculations, indicating that sulfur contributes substantially to the overall electronic structure of the system. Consequently, the introduction of S vacancies via NaOH etching can significantly modulate the electronic properties of the catalyst. In the pristine system without S vacancies, the valence band maximum (VBM) is primarily composed of S-3p orbitals hybridized with Zn-3d and In-4d orbitals. In contrast, the conduction band minimum (CBM) primarily consists of In-4d and Zn-3d orbitals, exhibiting typical semiconductor characteristics with the Fermi level positioned near the mid-gap. Upon introduction of S vacancies, new defect states emerge near the Fermi level (−1 to 0 eV), predominantly originating from the dangling bonds of Zn and In atoms adjacent to the vacancies. These defect states reduce the effective bandgap, thereby enhancing visible-light absorption, providing additional transition pathways for photogenerated electrons, and lowering the excitation energy. Moreover, these states can trap electrons, suppress electron-hole recombination, and consequently improve the photocatalytic efficiency.

Specifically, in the context of H_2_O_2_ production, S vacancies contribute via two mechanisms: (1) in the reduction pathway, the defect states act as electron traps, facilitating the reduction of O_2_ to form •O_2_^−^ intermediates; (2) in the oxidation pathway, the localized states at the VBM promote hole migration to the surface, enabling participation in oxidation reactions [46,47,48]. However, excessive S vacancies may form deep-level recombination centers, thereby diminishing catalytic activity. Therefore, careful tuning of the S vacancy concentration optimizes the electronic structure and photocatalytic performance of ZnIn_2_S_4_, providing a theoretical basis for efficient H_2_O_2_ production.

Using DFT calculations, we systematically investigated the ORR reaction mechanism of 4-SDZIS catalysts containing sulfur vacancies for H_2_O_2_ production. The analysis encompasses both the reaction pathway and the corresponding Gibbs free energy changes for each step. The ORR pathway for H_2_O_2_ synthesis typically involves four key steps [Equations (1)–(4)]:

Step 1: ∗ + O_2_ → ∗O_2_ (1)

Step 2: ∗O_2_ + e^−^ → ∗ + •O_2_^−^ (2)

Step 3: •O_2_^−^ + H^+^ → •OOH (3)

Step 4: •OOH + H^+^ + e^−^ → H_2_O_2_ (4)

Figure 5f presents the Gibbs free energy diagrams for 4-ZIS and 4-SDZIS. The first step corresponds to the adsorption of O_2_ on the catalyst surface. Thermodynamically, 4-ZIS exhibits a stronger adsorption energy (~−1.68 eV) compared to the much weaker adsorption on 4-SDZIS (~−0.05 eV), indicating that O_2_ binds more firmly to 4-ZIS. However, this strong adsorption hinders O_2_ desorption, limiting the formation of superoxide radicals (•O_2_^−^). Following adsorption, ∗O_2_ spontaneously accepts an electron to form •O_2_^−^ (step 2). O_2_ desorption is the rate-determining step (RDS) of the overall ORR, underscoring the importance of lowering the desorption energy to optimize catalytic performance. Subsequently, •O_2_^−^ reacts with a proton to form the hydroxyl radical (•OOH), which further reacts with another proton and electron to generate H_2_O_2_. The final two steps occur in solution, representing homogeneous reactions with inherently fast kinetics. In summary, 4-ZIS, lacking S vacancies, exhibits higher intermediate stability and larger energy barriers. In contrast, the introduction of S vacancies in 4-SDZIS reduces the energy required for O_2_ desorption, optimizes the photocatalytic H_2_O_2_ production pathway, and enhances reaction efficiency, which underlies the observed improvement in H_2_O_2_ yield.

## 4. Materials and Methods

### 4.1. Reagents

Zinc acetate dihydrate (Zn(CH_3_COO)_2_·2H_2_O, 99.99%), indium chloride (InCl_3_, 99.99%), and thioacetamide (TAA, 99.0%) were purchased from Shanghai Aladdin Biotechnology Co., Ltd., Shanghai, China. Ethylene glycol (EG, 99.0%, AR) and ammonium molybdate tetrahydrate ((NH_4_)_6_Mo_7_O_24_·4H_2_O, 99.9%) were obtained from Shanghai Macklin Biochemical Technology Co., Ltd., Shanghai, China. Citric acid (CA, 99.5%) was supplied by Maclean’s Reagent Co., Ltd., Shanghai, China). Sodium hydroxide (NaOH, 99%) and potassium iodide (KI, 99.0%) were purchased from Sinopharm Chemical Reagent Co., Ltd., Shanghai, China, and anhydrous ethanol (99.7%, AR) was provided by Shanghai Titan Technology Co., Ltd., Shanghai, China. All chemicals were of analytical grade and used as received without further purification. Deionized water with a resistivity of 18.25 MΩ·cm^−1^ was used throughout the experiments.

### 4.2. Synthesis of Photocatalysts

The ZIS and SDZIS samples were synthesized via a simple one-step hydrothermal method. In a typical procedure, 0.5 mmol of Zn(CH_3_COO)_2_·2H_2_O, 1 mmol of InCl_3_, 1 mmol of CA, and 3 mmol of NaOH were dissolved in 25 mL of deionized water containing 5 mL of EG. The mixture was vigorously stirred at room temperature for 30 min, after which 4 mmol TAA was added. Following an additional 30 min of stirring, the resulting heterogeneous solution was transferred into a 50 mL Teflon-lined stainless-steel autoclave and heated at 120 °C for 12 h. After natural cooling to room temperature, the obtained product (SDZIS) was collected by centrifugation, washed twice with anhydrous ethanol and deionized water, and then dried to yield a bright yellow powder (Figure S12).

Scheme 2 illustrates the synthesis scheme for SDZIS with sulfur vacancies. CA, acting as a chelating agent, coordinated with metal ions after protonation, effectively slowing the reaction kinetics and thereby inhibiting the rapid growth of ZIS nanosheets. A series of SDZIS samples was prepared with varying molar amounts of TAA (1, 2, 4, 6, and 8 mmol) and denoted as 1-SDZIS, 2-SDZIS, 4-SDZIS, 6-SDZIS, and 8-SDZIS, respectively. For comparison, ZIS was synthesized under identical conditions but without the addition of CA and NaOH.

### 4.3. Characterization and Theoretical Computation

The surface morphology of the samples was examined using a field-emission scanning electron microscope (FE-SEM, Quanta 250, FEI, Hillsboro, OR, USA). Microstructural features and elemental distributions were further characterized by transmission electron microscopy (TEM, Tecnai G2 F20, FEI, Hillsboro, OR, USA) equipped with energy-dispersive X-ray spectroscopy (EDS). The specific surface area was determined by N_2_ adsorption–desorption isotherm measurements using a 3-Flex physisorption analyzer (Micromeritics, Norcross, GA, USA), and calculated according to the Brunauer–Emmett–Teller (BET) method. The crystal structure was analyzed by X-ray diffraction (XRD, D8 Advance, Bruker, Karlsruhe, Germany) using Cu Kα radiation (λ = 0.15406 nm) over a 2θ range of 10–80°. The surface chemical states were identified using X-ray photoelectron spectroscopy (XPS, ESCALAB 250Xi, Thermo Fisher Scientific, Waltham, MA, USA), which employed monochromatic Al Kα radiation (hν = 1486.6 eV).

Optical absorption properties were investigated using a UV-Vis diffuse reflectance spectrophotometer (UV-3600i Plus, Shimadzu, Kyoto, Japan) in the wavelength range of 200–800 nm. Fourier-transform infrared (FT-IR) spectra were recorded on a Nicolet 5700 spectrometer (Thermo Fisher Scientific, Waltham, MA, USA) using the KBr pellet method within a scanning range of 4000–400 cm^−1^. The concentration of H_2_O_2_ produced in the reaction system was quantitatively determined with a UV-Vis spectrophotometer (Model 752N Plus, Shanghai Yidian Analytical Instrument Co., Ltd., Shanghai, China).

Electrochemical measurements were performed on a CHI760E electrochemical workstation (Chenhua Instruments, Shanghai, China) using a conventional three-electrode configuration. The as-prepared ZIS and SDZIS samples served as the working electrodes, a platinum wire was used as the counter electrode, and an Ag/AgCl electrode acted as the reference electrode. A 0.05 M Na_2_SO_4_ aqueous solution was employed as the electrolyte. Transient photocurrent (i-t) responses were recorded under 30 s light on/off cycles to evaluate the charge-separation behavior. Electrochemical impedance spectroscopy (EIS) was performed in a 50 mM Na_2_SO_4_ solution over a frequency range of 10^4^–10^−2^ Hz, using a 5 mV AC perturbation. Mott-Schottky (M-S) analysis was conducted in the dark to determine the semiconductor type and flat-band potential. All photoelectrochemical tests were performed under illumination from a 300 W xenon lamp (Perfect Light Technology, Beijing, China) equipped with a 420 nm cutoff filter, ensuring visible-light irradiation.

First principles calculations were employed to investigate the structural and photocatalytic mechanisms of H_2_O_2_ formation on ZnIn_2_S_4_, using the Gaussian Plane Wave (GPW) framework implemented in CP2K version 2024.3 [49,50]. The Perdew–Burke–Ernzerhof (PBE) functional [51] with D3 dispersion correction was adopted for geometry optimization at the DZVP-MOLOPT-SR-GTH basis-set level with Goedecker–Teter–Hutter (GTH) pseudopotentials. Total-energy calculations were refined using the more accurate TZV2P-MOLOPT-PBE-GTH basis set. The convergence criteria for structural optimization were set as follows: maximum force, 4.5 × 10^−4^ Hartree/Bohr; RMS force, 3.0 × 10^−4^ Hartree/Bohr; maximum displacement, 3.0 × 10^−3^ Bohr; and RMS displacement, 1.5 × 10^−3^ Bohr. The ZnIn_2_S_4_ unit cell was cleaved along the (1 0 2) plane to construct a 1 × 4 × 4 supercell. To eliminate interactions between adjacent layers, a vacuum spacing of 20 Å was introduced above the surface of the slab. All calculations were performed under two-dimensional periodic boundary conditions. Owing to the large supercell size and the correspondingly small Brillouin zone, all computations were conducted at the Γ point.

### 4.4. Hydrogen Peroxide Production Test Under Xenon Lamp

The photocatalytic activity of the samples was evaluated through H_2_O_2_ generation under visible-light irradiation using a 300 W xenon lamp (Beijing Bofeila Technology Co., Ltd., Beijing, China) equipped with a 420 nm cutoff filter. In a typical test, 10 mg of catalyst was dispersed in 50 mL of an aqueous ethanol solution (10 vol%) in a photocatalytic reactor, followed by ultrasonication for 5 min to ensure homogeneous dispersion. The reactor was then placed in a light-tight chamber, with the distance between the light source and the liquid surface maintained at approximately 10 cm. Ethanol (10 vol%) was intentionally introduced as a hole scavenger to consume photogenerated holes.

At designated irradiation intervals, 2 mL of the reaction solution was withdrawn using a syringe, and the catalyst was separated through a 0.22 μm membrane filter to obtain a clear filtrate. Subsequently, 500 μL of the filtrate was mixed with 2 mL of KI solution (0.1 mol L^−1^) and 50 μL of (NH_4_)_6_Mo_7_O_24_·4H_2_O solution (0.01 mol L^−1^), followed by a 10 min color-developing reaction. The absorbance of the resulting solution was measured at 352 nm using a UV-Vis spectrophotometer (Model 752N Plus, Shanghai Yidian Analytical Instrument Co., Ltd., Shanghai, China). The concentration of H_2_O_2_ was subsequently determined from the absorbance using a pre-established standard calibration curve.

## 5. Conclusions

In summary, we synthesized ZnIn_2_S_4_ with sulfur vacancies via a simple one-step hydrothermal method using CA as a metal-complexing agent and NaOH as an etching agent. Combined with DFT calculations, spectroscopic characterizations, and catalytic activity tests, we reveal that the H_2_O_2_ photosynthesis proceeds primarily through a radical-mediated pathway. Introducing S vacancies effectively modulates the adsorption strength of the catalyst toward reaction intermediates, and lowers the work function, thereby facilitating the formation of •O_2_^−^. As a result, the H_2_O_2_ production rate reached an impressive 2711.81 μmol g^−1^ h^−1^, approximately nine times higher than that of ZIS without S vacancies. This work presents an experimental–theoretical case study to understand how sulfur-deficiency-related electronic modulation correlates with photocatalytic H_2_O_2_ generation in ZnIn_2_S_4_-based systems.

## Data Availability

Data will be made available on request.

## Supplementary Materials

The following supporting information can be downloaded at: https://www.mdpi.com/article/10.3390/molecules31091512/s1, Figure S1: The XRD pattern of (a) X-ZIS and (b) X-SDZIS. Figure S2: (a) Zn 2p and (b) In 3d XPS spectra of 4-SDZIS. Figure S3: The Mott-Schottky (M-S) plot of 4-ZIS. Figure S4: XRD pattern of 4-SDZIS after six cycle tests. Figure S5: Long-term activity test of 4-SDZIS. Figure S6: Comparison of H_2_O_2_ production performance in the presence and absence of sacrificial agents. Figure S7: The RRDE polarization curves over ZIS and SDZIS at 1600 rpm O_2_-saturated 0.1 M KOH with ring current (upper part) and disk current (bottom part). Figure S8: The calculated average number of transferred electrons (n). Figure S9: The work function graphs of (a) ZnInS_2_ (Vs = 1) and (b) ZnInS_2_ (Vs = 3). Figure S10: The O_2_ adsorption capacity varies with the concentration of S vacancies. Figure S11: Differential charge density map. Figure S12: The synthesized X-ZIS and X-SDZIS with different TAA addition amounts. Figure S13: Photocatalytic reaction device. Table S1: Semi-quantitative XPS analysis of the X-SDZIS based on survey spectra. Table S2: The energy band parameters of all samples. References [23,26,29] are cited in the Supplementary Materials.
