# Supplementary material for: Sulfur Vacancies in ZnIn2S4 Boost Photocatalytic H2O2 Production: Unveiling the Role of Sulfur Vacancies in the Superoxide Radical Pathway for H2O2 Photosynthesis
Source: Molecules. 2026 May 2;31(9):1512. doi: 10.3390/molecules31091512 (PMC13164750; doi:10.3390/molecules31091512)
Supplement: Supplementary file 1 [file molecules-31-01512-s001.zip › molecules-4239870-supplementary.pdf]

Supplementary Material

Sulfur Vacancies in $ZnIn_2S_4$ Boost Photocatalytic H_2O_2 Production: Unveiling the Role of Sulfur Vacancies in the Superoxide Radical Pathway for H_2O_2 Photosynthesis

Boyi Ma, Degang Li *, Weimin Zhang and Siru Hao

School of Chemistry and Chemical Engineering, Shandong University of Technology, Zibo 255000, China; 19853352262@163.com (B.M.); wmzhang@sdut.edu.cn (W.Z.)
* Correspondence: ldg@sdut.edu.cn

1. Experimental section

1.1. Electrochemical measurements

Electrochemical measurements were performed using a CHI760E electrochemical workstation (CH Instruments, Shanghai, China). A standard three-electrode system was employed, where the as-prepared 4-ZIS and 4-SDZIS samples served as the working electrodes, with a platinum wire as the counter electrode and an Ag/AgCl electrode (saturated KCl solution) as the reference electrode. The electrolyte was a 0.05 M Na_2SO_4 aqueous solution, and all tests were conducted at room temperature. Electrode preparation procedure: 5 mg of catalyst powder was precisely weighed and dispersed in 1 mL of 70% (v/v) ethanol aqueous solution, followed by the addition of 10 μL Nafion solution (5 wt%). The mixture was ultrasonicated for 30 min to ensure homogeneous dispersion. An appropriate amount of the suspension was then uniformly coated onto a pre-treated glassy carbon electrode (3 mm in diameter) and dried at room temperature for subsequent use.

1.2. Hydrogen peroxide (H_2O_2) detection experiment

This study employed a 100 mL photocatalytic reactor to conduct experiments on the photocatalytic synthesis of hydrogen peroxide (H_2O_2). The specific procedure was as follows: 10 mg of the photocatalyst was uniformly dispersed in 50 mL of an aqueous solution containing 10 vol% ethanol, and the photocatalytic reaction was carried out under irradiation from a 300 W xenon lamp (equipped with a 420 nm cutoff filter, $\lambda \geq 420$ nm). To systematically investigate the effects of different reaction conditions on H_2O_2 production performance, comparative experiments were designed with the following variations: (1) addition of different radical scavengers, and (2) introduction of different gas atmospheres (N_2 or air). Notably, during the dark reaction phase, N_2 or air was continuously purged into the reaction system for 1 hour to ensure complete removal of residual oxygen, thereby enabling an accurate evaluation of the influence of different gas environments on the photocatalytic H_2O_2 generation activity. All experiments were performed at room temperature.

The concentration of H_2O_2 in the reaction system was quantitatively determined using an iodometric method coupled with UV–Vis spectrophotometry (Shanghai Yidian Analytical Instruments Co., Ltd., model 752N plus). The detailed testing procedure was as follows: Every 15 minutes, 2 mL of the reaction solution was withdrawn and filtered through a 0.22 μm aqueous microporous membrane (MCE membrane). Then, 500 μL of the filtrate was precisely measured, followed by sequential addition of 2 mL of KI solution (0.1 mol/L) and 50 μL of ammonium molybdate solution ($(NH_4)_6Mo_7O_{24}\cdot4H_2O$, 0.01 mol/L). After a 10-minute reaction in the dark, the absorbance was immediately measured at a wavelength of 352 nm. This detection method is based on the following reaction mechanism: Under acidic conditions, H_2O_2 reacts with KI to form an I_3^- complex ($H_2O_2 + 3I^- + 2H^+ \rightarrow I_3^- + 2H_2O$), which exhibits a characteristic absorption peak at 352 nm. The absorbance shows a good linear relationship with H_2O_2 concentration, enabling accurate quantitative analysis of H_2O_2 through the establishment of a standard calibration curve.

1.3. Electrochemical measurement of oxygen reduction reaction (ORR)

The oxygen reduction reaction (ORR) performance of the 4-ZIS and 4-SDZIS samples was systematically evaluated using a standard three-electrode system. The experimental setup consisted of a rotating ring-disk electrode (RRDE, 400 rpm) as the working electrode, a Hg/HgO reference electrode, and a carbon rod counter electrode. The electrolyte was an O_2-saturated 0.1 M KOH solution. The working electrode was prepared as follows: First, 5 mg of the catalyst was uniformly dispersed in 1 mL of an aqueous solution containing 70% (v/v) ethanol. Then, 10 μL of Nafion solution (5 wt%) was added as a binder, followed by 30 min of ultrasonication to ensure a homogeneous catalyst ink. Finally, 8 μL of the ink was drop-casted onto the RRDE surface and dried at room temperature to obtain the working electrode.

The number of transferred electrons (n) is calculated according to the following formula:

$$n=I_d/(\ I_d+I_r/N)\times100\%$$

Where Ir is the ring current, Id is the disc current, and N is the collection efficiency (N=0.32).

1.4. SCC Efficiency measurements:

The solar-to-chemical energy conversion (SCC) efficiency was determined by using a 300W xenon lamp (100 mW cm^{-2}) as the light source. 10 mg of the catalyst and 50 ml of ethanol solution (10 vol%) were placed in a quartz light catalytic reactor, and air was continuously introduced into the reactor during the experiment. The SCC efficiency was calculated via the following equation:

$$SCC=\frac{[\Delta G_{H_2O_2}]\times[n_{H_2O_2}]}{I\times S\times T}\times100\%$$

Where ΔG=117 kJ mol^{-1}. For instance, when using 4-SDZIS as the catalyst, during the 1 h irradiation period, the irradiated sample area was 3.85 cm^2. Therefore, the calculated total input energy (J) is 1440 J. During the photocatalytic reaction process, 27.11 μmol of H_2O_2 was produced within 1 hour, and the energy generated by the photocatalytic H_2O_2 was 3.17 J. Consequently, the SCC efficiency can be calculated as follows:

$$SCC=\frac{[\Delta G_{H_2O_2}]\times[n_{H_2O_2}]}{I\times S\times T}\times100\%=\frac{117\times10^{3}\times27.11\times10^{-6}}{100\times3.85\times10^{-3}\times3600}\times100\%=0.23\%$$

2. Supplementary Figures

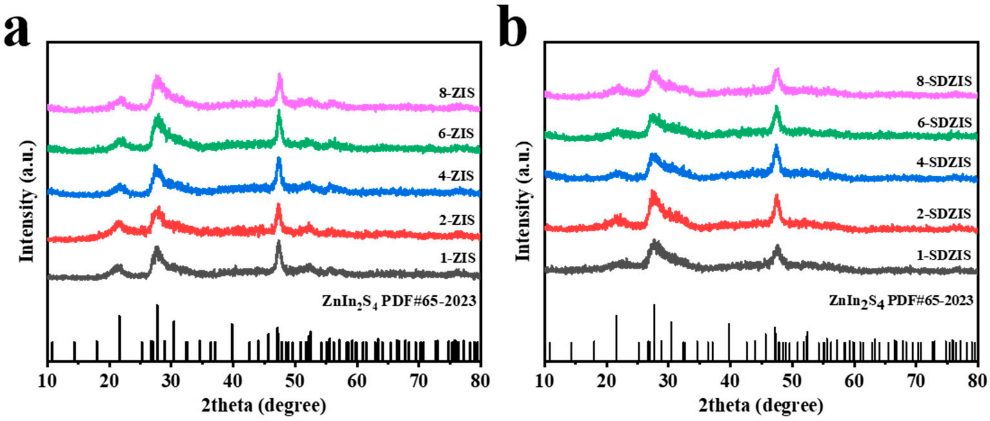

Figure S1. The XRD pattern of (a) X-ZIS and (b) X-SDZIS.

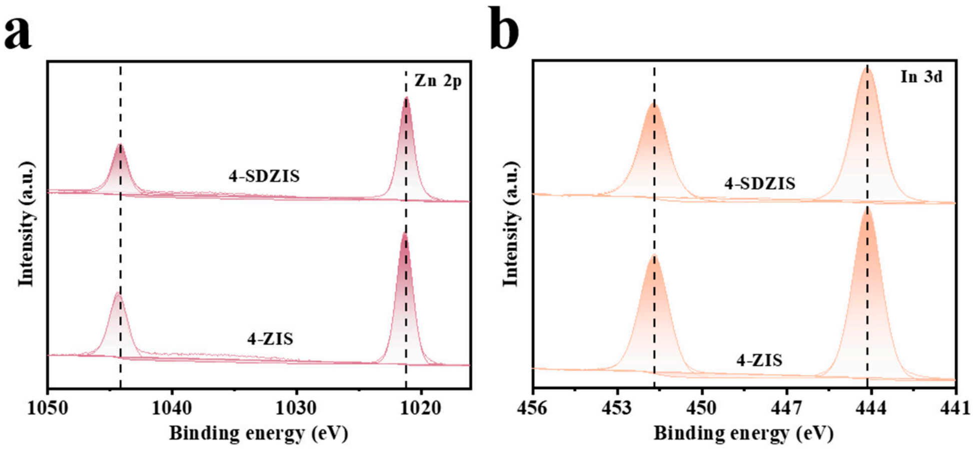

Figure S2. (a) Zn 2p and (b) In 3d XPS spectra of 4-SDZIS.

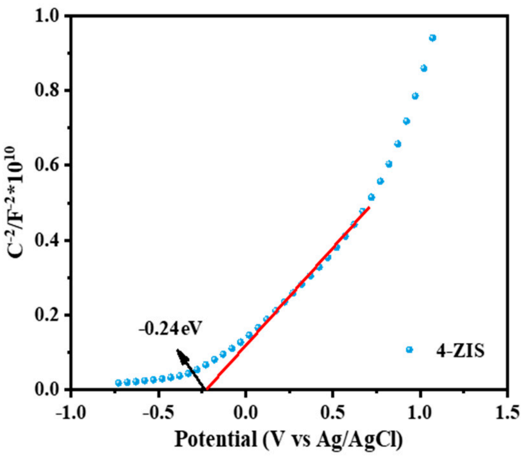

Figure S3. The Mott–Schottky (M-S) plot of 4-ZIS.

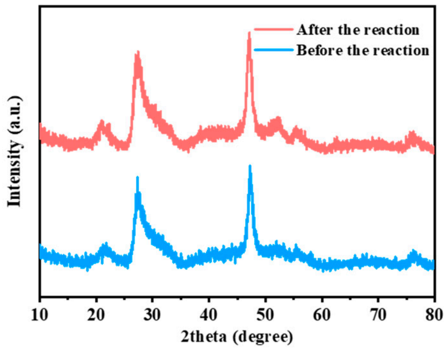

Figure S4. XRD pattern of 4-SDZIS after six cycle tests.

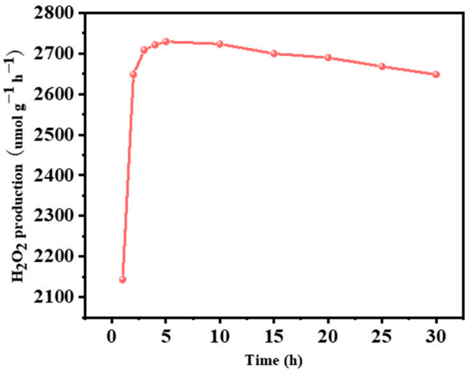

Figure S5. Long-term activity test of 4-SDZIS

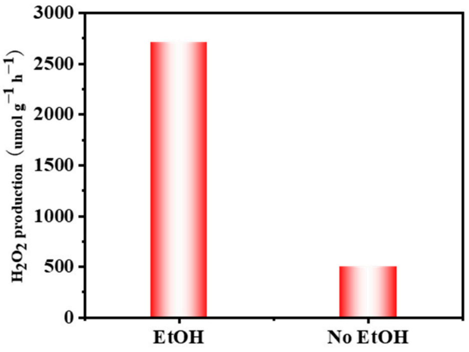

Figure S6. Comparison of H_2O_2 production performance in the presence and absence of sacrificial agents.

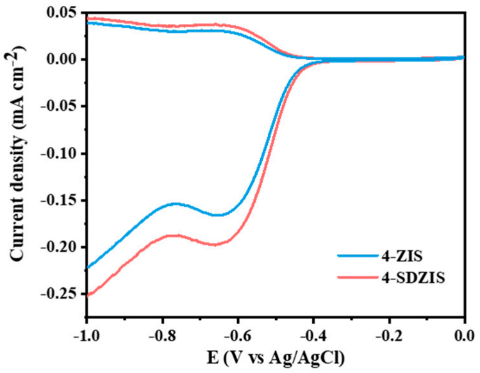

Figure S7. The RRDE polarization curves over ZIS and SDZIS at 1600 rpm O_2-saturated 0.1 M KOH with ring current (upper part) and disk current (bottom part).

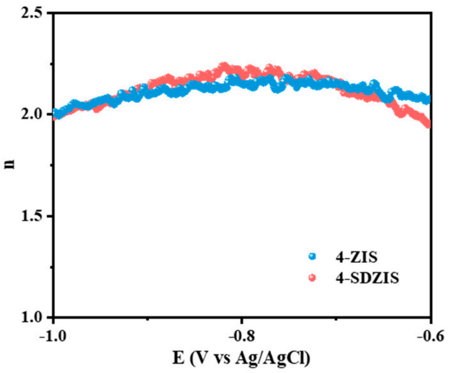

Figure S8. The calculated average number of transferred electrons (n).

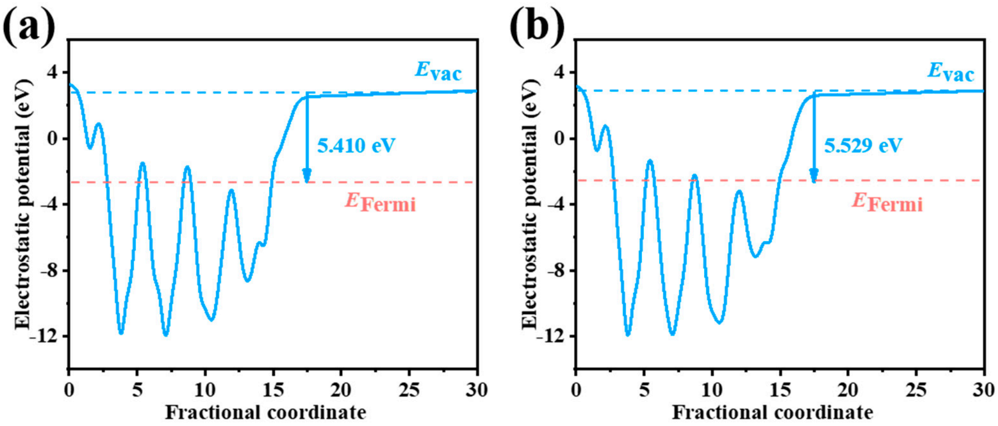

Figure S9. The work function graphs of (a) $ZnInS_2$ (Vs = 1) and (b) $ZnInS_2$ (Vs = 3).

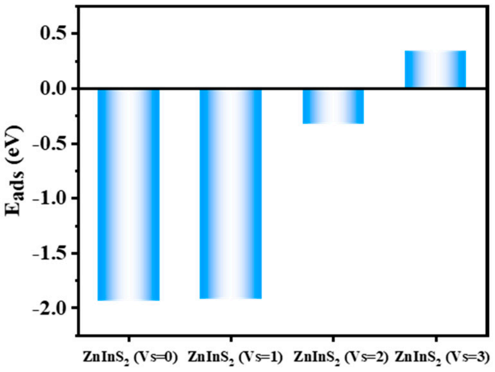

Figure S10. The O_2 adsorption capacity varies with the concentration of S vacancies.

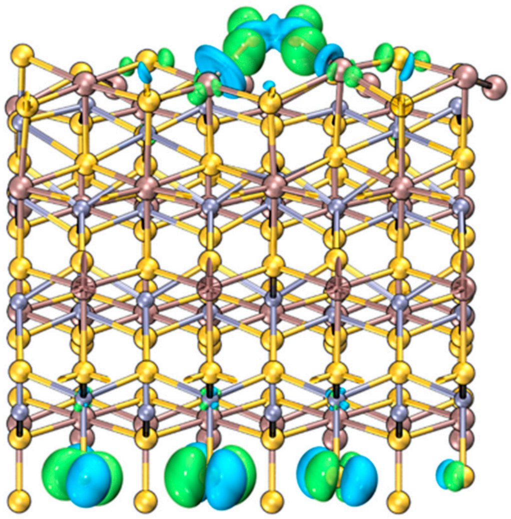

Figure S11. Differential charge density map

In the differential charge density plot, green indicates an increase in electrons, while blue indicates a decrease in electrons. As can be seen from the figure, the electrons on the catalyst have significantly transferred to the O_2 molecules.

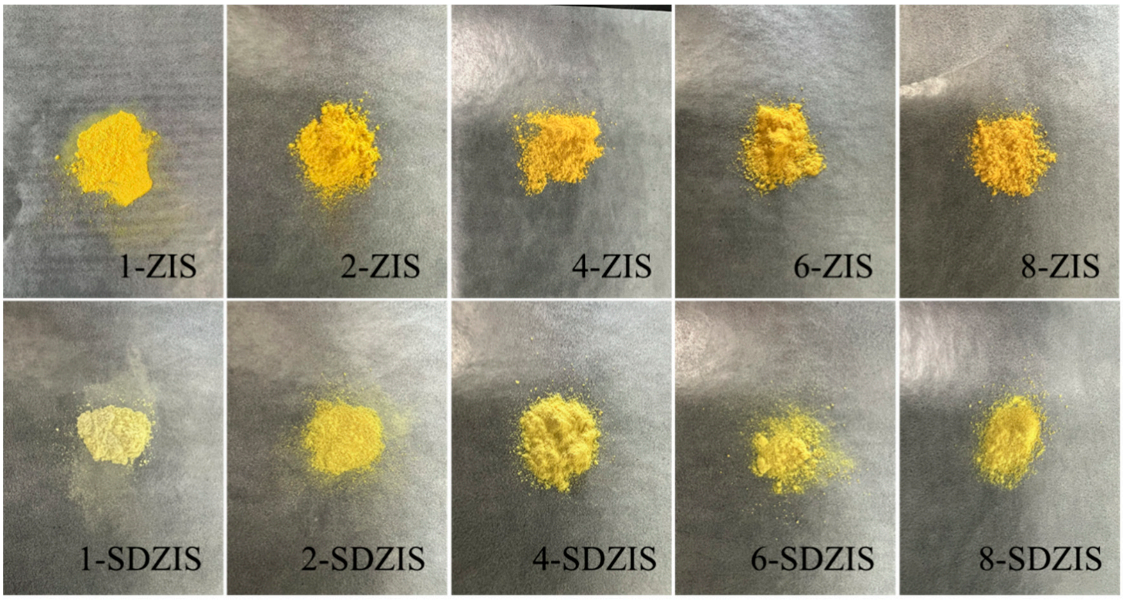

Figure S12. The synthesized X-ZIS and X-SDZIS with different TAA addition amounts.

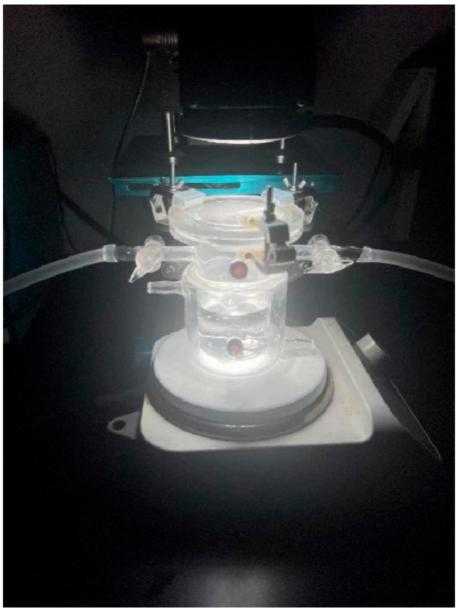

Figure S13. Photocatalytic reaction device.

Table S1. Semi-quantitative XPS analysis of the X-SDZIS based on survey spectra.

Sample	S vacancy content (%)
2-SDZIS	4.4
4-SDZIS	3.1
8-SDZIS	2.2

Table S2. The energy band parameters of all samples.

Sample	E_f (vs. NHE)	VB (vs. NHE)	CB (vs. NHE)	Eg
4-ZIS	-0.24	2.38	-0.14	2.52
4-SDZIS	-0.43	2.16	-0.33	2.49

References

1.→ H. Peng, H. Yang, J. Han, X. Liu, D. Su, T. Yang, S. Liu, C. Pao, Z. Hu, Q. Zhang, Y. Xu, H. Geng, X. Huang, Defective ZnIn2S4 nanosheets for visible-light and sacrificial-agent-free H2O2 photosynthesis via O2/H2O redox, J. Am. Chem. Soc. 50 (2023), https://doi.org/10.1021/jacs.3c10390.

2.→ K. Zhang, M. Dan, J. Yang, F. Wu, L. Wang, H. Tang, Z. Liu, Surface energy mediated sulfur vacancy of ZnIn2S4 atomic layers for photocatalytic H2O2 production, 33 (2023) 2302964, https://doi.org/10.1002/adfm.202302964.

3.→ Y. Kong, D. Li, C. Zhang, W. Han, Y. Xue, W. Zhang, H. Sun, S. Wang, X. Duan, Synergistic silver doping and N vacancy promoting photocatalytic performances of carbon nitride for pollutant oxidation and hydrogen production, Chem. Eng. J. 479 (2024) 147676, https://doi.org/10.1016/j.cej.2023.147676.
